# PARP inhibitors in platinum-sensitive high-grade serous ovarian cancer

**DOI:** 10.1007/s00280-018-3532-9

**Published:** 2018-02-20

**Authors:** Robert D. Morgan, Andrew R. Clamp, D. Gareth R. Evans, Richard J. Edmondson, Gordon C. Jayson

**Affiliations:** 1 Department of Medical Oncology, The Christie NHS Foundation Trust, Manchester, UK; 2 Manchester Cancer Research Centre, The University of Manchester, Wilmslow Road, Manchester, M20 4BX UK; 3 Division of Evolution and Genomic Sciences, Manchester Academic Health Science Centre, University of Manchester, Manchester, UK; 4 Department of Obstetrics and Gynaecology, St Mary’s Hospital, Central Manchester NHS Foundation Trust, Manchester, UK; 5 Division of Cancer Sciences, Faculty of Biology, Medicine and Health, University of Manchester, Manchester, UK

**Keywords:** Ovarian cancer, Platinum sensitivity, PARP inhibitors, BRCA mutation

## Abstract

**Purpose:**

Poly(ADP-ribose) polymerase inhibitors (PARPi) have changed the management of high-grade serous ovarian cancer (HGSOC). The rationale for the development of PARPi was based on the concept of synthetic lethality, in which a cell can survive a deficiency of one gene/gene product, but may die if there is a deficiency in a combination of genes/gene products. In women with *BRCA1*/*2* deficiency within their ovarian cancer tissue, inhibition of PARP imposes an intolerable burden of DNA damage repair deficiency and may induce cell death.

**Methods:**

Clinical trials have evaluated PARPi as single-agent therapeutics and as maintenance treatment following platinum-based chemotherapy for HGSOC. Clinical data suggest the most impressive anti-tumour activity occurs in women with platinum-sensitive ovarian cancer and germline or somatic *BRCA1*/*2* mutations (g/s*BRCA*mt).

**Results:**

In the maintenance setting, randomised trials have shown that PARPi compared to placebo reduce the hazard ratio for the development of progressive disease to 0.2–0.27 for patients with a g/s*BRCA*mt; to 0.34–0.38 for patients with putative evidence of DNA damage repair deficiency; and to 0.35–0.45 in an unselected population with HGSOC. Furthermore, phase 1/2 trials have reported single-agent anti-tumour response rates in g*BRCA*mt of approximately 50% in platinum-sensitive and 25% in platinum-resistant disease.

**Conclusion:**

Here, we discuss the evidence for the use of PARPi as single-agent therapeutics and maintenance treatment in HGSOC and evaluate the genetic assays used in clinical trials so far. We discuss the emerging role of platinum sensitivity as a broad eligibility criteria for the use of PARPi.

## Introduction

Epithelial ovarian cancer is the fourth commonest cause of female cancer-related death in the west [1]. The commonest histological subtype, high-grade serous carcinoma, accounts for approximately 70% of cases [1]. Epithelial ovarian cancer often presents with advanced disease. Primary treatment is tailored to individual patients but often involves cytoreductive surgery followed by platinum-based chemotherapy. In a minority of patients with advanced stage disease, this approach is associated with long-term survival, but sadly the majority will develop recurrent disease within 12–18 months of their primary treatment.

Upon disease relapse, treatment switches to controlling symptoms and improving survival with sequential lines of platinum- and/or taxane-based chemotherapy, often resulting in diminishing disease-free intervals until drug resistance develops and palliation is required. The 5-year survival for ovarian cancer is approximately 35% [2]. Two drug classes of maintenance therapy have so far been developed [3]. The first group is the VEGF inhibitors, which largely improve progression-free survival (PFS). Improvements in overall survival (OS) have only been reported in occasional trials or trial subgroups [4].

The second class of maintenance therapy agents are the poly(ADP-ribose) polymerase inhibitors (PARPi) [5]. These small molecule inhibitors exploit the susceptibility of cancer cells to defects in DNA damage repair and have shown impressive results in clinical trials as both single-agent therapeutics and maintenance treatments. Herein, we discuss the use of PARPi in high-grade serous ovarian carcinoma and the genetic assays that have been assessed as potential predictive biomarkers.

### Homologous recombination repair

Following the initial description of the sensitivity of *BRCA*-deficient cells to PARPi [6, 7], further work tested the hypothesis that impairments in double-strand DNA repair might predict for sensitivity to PARPi. Homologous recombination (HR) repair is a high-fidelity genetic recombination process involved in the repair of DNA double-strand breaks (DSBs) [8]. It occurs during the G2/S phase of the cell cycle where the presence of a sister chromatid/homologous chromosome allows error-free repair of DSBs and preservation of genomic integrity [8]. Germline loss-of-function (LOF) monoallelic mutations in genes involved in HR repair such as *BRCA1, BRCA2, RAD51C, RAD51D* and *BRIP1* are associated with an increased risk of developing familial ovarian cancer [9–15]. For carriers of the high-penetrance cancer-susceptibility genes *BRCA1* and *BRCA2*, the life-time risk of ovarian cancer, and particularly high-grade serous carcinoma, is between 40–70 and 20–50%, respectively [16, 17]. Malignant transformation in *BRCA*-related familial ovarian cancer is thought to occur when the remaining wild-type allele undergoes pathogenic mutation or epigenetic silencing, thereby leaving only the germline *BRCA1*/*2* mutant allele [18].

A deficiency in *BRCA1* or *BRCA2* within a cell is believed to impair HR repair, termed homologous recombination deficiency (HRD), and place a greater reliance upon alternative DNA repair pathways to repair DSBs, such as non-homologous end joining (NHEJ) and microhomology-mediated end joining (MMEJ) [19]. These alternative pathways are of low fidelity and error-prone, with potential for losses or gains of nucleotide bases within the cell’s genome during the repair process [20]. A reliance on NHEJ and/or MMEJ in cells with *BRCA1*/*2* deficiency increases the probability of mutations occurring within the genome, a process known as genomic instability, and may ultimately lead to malignant transformation [21]. The *BRCA1*/*2* genes are, therefore, considered tumour suppressor genes and are likely to require biallelic LOF for tumorigenesis to occur; in keeping with Knudson’s double-hit hypothesis [22–24]. In women with a somatic *BRCA1*/*2* mutation in the tumour tissue only, biallelic LOF occurs through mutations and/or epigenetic silencing in both wild-type alleles and is often clonal [25, 26]. The relationship between possessing a germline monoallelic mutation in other non-*BRCA* HR repair genes and genomic instability/tumorigenesis is less clear.

### PARP inhibitors

PARP are a family of cellular enzymes that are involved in a variety of biological functions. The most abundant and well-characterised members are PARP-1 and PARP-2, which have a role in DNA damage detection and repair [27]. Within their catalytic site, nicotinamide adenine dinucleotide is used as a substrate to form polymers of ADP-ribose in a process called poly (ADP-ribosyl)ation or PARylation [28]. PARP binds to DNA at single-strand breaks (SSBs) and forms PAR on itself and other accessory proteins associated with DNA. These polymers are then able to recruit proteins involved in the base excision repair (BER) pathway; utilised to repair SSBs [29].

Small molecule inhibitors of PARP-1/2 were engineered to inhibit the catalytic domain at nanomolar concentrations. By inhibiting PARP, SSBs remain unrepaired and may potentially form lethal DSBs as the ensuing replication fork stalls and/or collapses [19]. More recent evidence also suggests that PARPi trap PARP on DNA and thereby prevent the dissociation required for the BER pathway to proceed [30–32]. There is also evidence to suggest that PARP is involved in NHEJ and that dysregulation of NHEJ may determine PARPi sensitivity [33, 34].

Preclinical work has convincingly demonstrated that cells deficient in *BRCA1*/*2* were sensitive to PARP inhibition. The proposed mechanism for this effect is synthetic lethality, in which a cell is able to survive with a deficiency in one gene/gene product but may die if a deficiency occurs in a particular combination of two or more genes/gene products [35, 36]. A deficiency in *BRCA1*/*2* is believed to impair a cell’s ability to repair DSBs through HR repair. A lethal second deficiency is brought about by targeted pharmacological inhibition of PARP-1/2 [19].

The use of PARPi is changing how high-grade serous ovarian carcinoma is treated. PARP inhibitors have been evaluated in two distinct clinical settings; as single-agent therapeutics and as maintenance treatment following a response to platinum-based chemotherapy.

## Single-agent therapy

PARPi single-agent therapy has been thoroughly investigated in phase 1/2 trials in women with ovarian cancer (see Table 1) [37–53]. These trials predominantly enrolled women with high-grade serous ovarian carcinoma and a germline *BRCA1*/*2* mutation (g*BRCA*mt). Most women had been pre-treated with several lines of traditional cytotoxic chemotherapy and in most trials platinum sensitivity was not mandated. A range of doses of individual PARPi were used in the search for recommended phase 2/3 doses. Nevertheless, data from these trials reported encouraging anti-tumour responses across all PARPi in selected groups of patients. Patients with g*BRCA*mt often had better anti-tumour responses than those with wild-type *BRCA1*/*2* (*BRCA*wt) and patients with platinum-sensitive disease had better responses than women with platinum-resistant or refractory disease. Data also suggested that women with platinum-resistant disease who carried a g*BRCA*mt achieved better objective response rates (ORRs) than those with *BRCA*wt.

**Table 1:** Objective response rates (ORR) as per RECIST (CR/PR) to PARPi single-agent therapy in ovarian cancer

Clinical trial	PARPi	Dose	*BRCA* status	ORR, % (95% CI)
Phase 1 trials				
Fong et al.^a^	Olaparib	200 mg BD^d^	g*BRCA*mt	Plat-Sen 46 Plat-Res 33
Mateo et al.^a^	Olaparib	300 mg BD^e^ 400 mg BD^e^	g*BRCA*mt	Plat-Sen & Plat-Res 38^g^ Plat-Sen & Plat-Res 42^g^
Kristeleit et al.	Rucaparib	40 mg OD − 840 mg BD	g*BRCA*mt	Plat-Sen 12 Plat-Res 9
Sandhu et al.^a^	Niraparib	300 mg OD	*BRCA*mt Sporadic HGSOC	Plat-Sen 50 Plat-Res 33 Plat-Sen 33 Plat-Res 5
De Bono et al.^a^	Talazoparib	0.025–1.1 mg/day OD	g*BRCA*mt	Plat-Sen 55 Plat-Res 20
Phase 2 trials				
Auden et al.	Olaparib	400 mg BD	g*BRCA*mt	Plat-Sen 38 Plat-Res 30
Gelmon et al.	Olaparib	400 mg BD	*BRCA*mt *BRCA*wt or unknown HGSOC	Plat-Sen 60 Plat-Res 33 Plat-Sen 50 Plat-Res 4
Kaye et al.^b^	Olaparib	400 mg BD	g*BRCA*mt	Plat-Sen & Plat-Res 31^g^
Kaufman et al.	Olaparib	400 mg BD	g*BRCA*mt	Plat-Res only^h^ 31 (25–38)
Drew et al.	Rucaparib	Variable oral dosing levels^f^	g*BRCA*mt	Plat-Sen & Plat-Res 21^g^
Kristeleit et al.	Rucaparib	600 mg BD	*BRCA*mt	Plat-Sen 60 (43–74)
ARIEL2 (Part 1)^b^	Rucaparib	600 mg BD	*BRCA*mt *BRCA*wt/LOH^High^ *BRCA*wt/LOH^Low^	Plat-Sen 80 (64–91) Plat-Sen 29 (20–40) Plat-Sen 10 (4–20)
Coleman et al.	Veliparib	400 mg BD	g*BRCA*mt	Plat-Sen 35 (18–56)^i^ Plat-Res 20 (9–36)^i^
Steffensen et al.^c^	Veliparib	300 mg BD	g*BRCA*mt	Plat-Sen 56 Plat-Res 45

*BRCAmt* pathogenic *BRCA*1/2 mutation, *BRCAwt* wild type *BRCA*1/2, *BD* twice daily, *CR* complete response, *HGSOC* high-grade serous ovarian cancer, *LOH* loss of heterozygosity, *n* number of patients, *OD* once daily, *PR* partial response, *Plat-Sen* platinum sensitivity, *Plat-Res* platinum resistant

^a^Phase 1 dose expansion study

^b^Randomised phase 2 trial

^c^Phase 1/2 study. The ORR for Phase 2 are included in this table

^d^The majority of patients (*n* = 39/50) received 200 mg twice daily. Dose levels ranged from 40 mg once daily for 2 out of every 3 weeks to 600 mg twice daily

^e^ORR for the tablet formulation (150 mg tablet), as opposed to capsule formulation, is reported in this table

^f^Only patients receiving oral rucaparib included in this table; oral doses ranged from 92 mg once daily for 7 days q21 days to 600 mg twice daily continuous daily dosing

^g^Combined ORR for patients with platinum-sensitive or platinum-resistant disease

^h^Study included women with platinum-resistant disease and those not suitable for further platinum therapy

^i^90% confidence interval

The most impressive anti-tumour response reported was in the phase 2, single arm, multi-centre trial, ARIEL2 (Part 1) in which women with a germline or somatic *BRCA*mt (g/s*BRCA*mt) achieved a RECIST ORR [complete/partial response (CR/PR)] of 80% (95% CI 62–97%) with rucaparib single-agent therapy [53]. The vast majority of women recruited had high-grade serous ovarian cancer and all women had a progression-free interval following the penultimate platinum-based chemotherapy of at least 6 months. The median number of prior lines of chemotherapy was 2 (inter quartile range [IQR] of 1 to 2). In the same trial, women with *BRCA*wt had a more modest, but encouraging, RECIST ORR (CR/PR) of 21% (the objective response by combined RECIST and CA-125 was 35%).

Across all phase 1/2 trials investigating PARPi as a single-agent therapy women with platinum-sensitive ovarian cancer achieved better ORRs compared to those with platinum-resistant/refractory disease (see Table 1). In addition, a number of trials demonstrated that a longer platinum-free interval (PFI) correlated with a higher likelihood of achieving an anti-tumour response [41, 43, 47, 54].

Data from a number of phase 1/2 trials also suggested that in the platinum-resistant setting, eligibility for PARPi single-agent therapy might appropriately include *BRCA1*/*2* mutational status. Indeed, the ORRs for women with a g*BRCA*mt and platinum-resistant disease were noticeably higher (approximately 20–30%) across all PARPi than that seen in women with *BRCA*wt (see Table 1). In a phase-1 dose expansion trial assessing niraparib, Sandhu et al. reported an ORR of 5% in women with platinum-resistant sporadic high-grade serous ovarian or primary peritoneal cancer compared to 33% in women who had a g*BRCA*mt [51]. In addition, in a non-randomised phase 2 trial assessing olaparib, Gelmon et al. reported an ORR as per RECIST (CR/PR) of 33% in women with a g*BRCA*mt and platinum-resistant high-grade serous or undifferentiated ovarian carcinoma, but a much lower ORR of 4% in those with *BRCA*wt [44]. Interestingly, in the same trial Gelmon et al. also showed that all women with a g*BRCA*mt and platinum-resistant disease achieved a CA-125 treatment response compared to only 40% with *BRCA*wt. These data may suggest that the acquisition of platinum-resistance in women with *BRCA*wt is associated with PARPi single-agent therapy resistance, but that the presence of a g*BRCA*mt maintains PARPi sensitivity. Women with *BRCA*wt and platinum-resistant disease may, therefore, benefit more from combinational therapy with PARPi and additional targeted treatments, anti-angiogenic inhibitors and/or immune therapy [55–57].

As single agents, PARPi brought about modest and manageable toxicities. Specific grade 3–4 adverse events (AEs) included most commonly anaemia but also fatigue, nausea and vomiting. These AEs are now recognised class effects of which the majority are grade 1–2. Rucaparib caused a noticeable comparable increase in grade 3–4 ALT/AST (> 10%) although these increases often occurred within the first few weeks after starting treatment and were not associated with any symptoms, infrequently led to treatment discontinuation (< 1%), and, reversed following dose omission [58]. The recommended phase 2/3 dose of niraparib (300 mg once daily) did cause notable myelosuppression [51]. Rare (≤ 1%) but important class-specific AEs also included myelodysplastic syndrome/acute myeloid leukaemia (MDS/AML) and pneumonitis. The incidence of MDS/AML was similar across all PARPi and did not depend upon duration of treatment [37, 45, 51, 54].

A number of trials are ongoing to assess PARPi as single-agent therapy in the platinum-sensitive and platinum-resistant setting (see Table 2).

**Table 2:** Ongoing phase 2/3 trials assessing PARPi as single-agent therapy and maintenance monotherapy

Single-agent therapy	
SOLO-3 (NCT02282020)	Olaparib vs. chemotherapy (g*BRCA*mt, platinum-sensitive)
OCTOVA (NCT03117933)	Olaparib ± cediranib vs. chemotherapy (g/s *BRCA*mt, platinum-resistance)
QUADRA (NCT02354586)	Niraparib after 3 or 4 lines of chemotherapy (single arm, phase 2)
ARIEL2 Part 2 (NCT01891344)	Rucaparib after ≥ 3 lines of chemotherapy (single arm, phase 2)
ARIEL4 (NCT02855944)	Rucaparib vs. chemotherapy (g/s *BRCA*mt, platinum-sensitive or resistance)

Maintenance monotherapy treatment	
SOLO-1 (NCT01844986)	Olaparib vs. placebo (g/s*BRCA*mt, following response to first-line platinum therapy)
OReO (NCT03106987)	Olaparib vs. placebo (re-challenge maintenance olaparib monotherapy after response to repeat platinum therapy)
PRIMA (NCT02655016)	Niraparib vs. placebo (following response to first-line platinum-based therapy)
GOG3005 (NCT02470585)	Veliparib vs. placebo (following concurrent first-line carboplatin, paclitaxel & veliparib)

*g* germline, *g/s* germline or somatic

## Maintenance therapy

More recently, the utilisation of PARPi has focused on maintenance treatment following a response to platinum-based chemotherapy. Several phase 2/3 trials have assessed PARPi as maintenance treatment in women with relapsed high-grade ovarian cancer [59–63] (see Table 3). In each trial, PARPi were commenced following a treatment response [RECIST (CR/PR) ± CA-125 response] to platinum therapy. These trials primary outcome was progression-free survival (PFS); either investigator assessed or blinded independent central review (BICR; see Table 3). In addition, secondary efficacy outcomes included time to first subsequent therapy (TFST) and time to second subsequent therapy (TSST) as there was concern that platinum-resistance may be induced by PARPi and so these exploratory endpoints allow analysis of this (see Table 4).

**Table 3:** Progression-free survival (PFS) in phase 2/3 trials assessing PARPi as maintenance treatment

Clinical Trial	Subgroups	Study arms	PFS/months (95% CI)	HR (95% CI)	*P value*
Study 19^a^	HGSOC	Olaparib vs. Placebo	8.4 vs. 4.8	0.35 (0.25–0.49)	< 0.001
	g/s*BRCA*mt	Olaparib vs. Placebo	11.2 (8.3-NC) vs. 4.3 (3.0-5.4)	0.18 (0.10–0.31)	< 0.0001
SOLO2^a^	g*BRCA*mt	Olaparib vs. Placebo	19.1 (16.3–25.7) vs. 5.5 (5.2–5.8)	0.30 (0.22–0.41)	< 0.0001
NOVA^b^	g*BRCA*mt	Niraparib vs. Placebo	21.0 vs. 5.5	0.27 (0.17–0.41)	< 0.001
	Non-g*BRCA*mt /HRD-carcinoma	Niraparib vs. Placebo	12.9 vs. 3.8	0.38 (0.24–0.59)	< 0.001
	Non-g*BRCA*mt	Niraparib vs. Placebo	9.3 vs. 3.9	0.45 (0.34–0.61)	< 0.001
ARIEL3^a^	g/s*BRCA*mt	Rucaparib vs. Placebo	16.6 (13.4–22.9) vs. 5.4 (3.4–6.7)	0.23 (0.16–0.34)	< 0.0001
	HRD carcinoma	Rucaparib vs. Placebo	13.6 (10.9–16.2) vs. 5.4 (5.1–5.6)	0.32 (0.24–0.42)	< 0.0001
	ITT population	Rucaparib vs. Placebo	10.8 (8.3–11.4) vs. 5.4 (5.3–5.5)	0.36 (0.30–0.45)	< 0.0001

*CI* confidence interval, *g*/*sBRCAmt* germline or somatic, *HGSOC* high-grade serous ovarian carcinoma, *HRD* Homologous Recombination Deficiency, *ITT* intention to treat, *NC* not calculable

^a^Investigator assessed PFS

^b^Blinded independent central review PFS

**Table 4:** Median time to first subsequent therapy (TFST) and time to second subsequent therapy (TSST) reported in phase 2/3 trials assessing PARPi as maintenance treatment

Clinical trial	Subgroups	Study arms	TFST/months (95% CI)	TSST/months (95% CI)
Study 19	HGSOC	Olaparib vs. Placebo	13.4 (11.3–15.7) vs. 6.7 (5.7–8.2) HR 0.39 (0.29–0.51) *P* < 0.0001	19.1 (16.5–22.0) vs. 14.8 (14.0-17.2) HR 0.52 (0.39–0.68) *P* < 0.0001
	g/s*BRCA*mt	Olaparib vs. Placebo	15.6 (12.3–28.2) vs. 6.2 (5.3–9.2) HR 0.32 (0.22–0.48) *P* < 0.0001	22.0 (17.7–34.9) vs. 15.3 (14.0-18.7) HR 0.41 (0.28–0.62) *P* < 0.0001
SOLO2	g*BRCA*mt	Olaparib vs. Placebo	27.9 (22.6-NC) vs. 7.1 (6.3–8.3) HR 0.28 (0.21–0.38) *P* < 0.0001	NR (NC) vs. 18.2 (15.0-20.5) HR 0.37 (0.26–0.53) *P* < 0.0001
NOVA	g*BRCA*mt	Niraparib vs. Placebo	21 (17.5-NR) vs. 8.4 (6.6–10.6) HR 0.31 (0.21–0.48) *P* < 0.001	Not reported
	Non-g*BRCA*mt	Niraparib vs. Placebo	11.8 (9.7–13.1) vs. 7.2 (5.7–8.5) HR 0.55 (0.41–0.72) *P* < 0.001	Not reported

*CI* confidence interval, *HR* Hazard Ratio, *NR* not reached, *NC* not calculable

### Randomised phase 2 maintenance therapy trial

Study 19, a randomised, placebo-controlled, phase 2 trial, first assessed PARPi as maintenance treatment in ovarian cancer (see Table 3) [60]. All enrolled women had recurrent, platinum sensitive (defined as a progression-free interval of 6 months or more from the penultimate platinum chemotherapy) high-grade serous ovarian carcinoma and had achieved a CR/PR to their latest platinum-based chemotherapy. Patients were randomised to receive either olaparib 400 mg twice daily (50 mg capsules) or placebo. The median number of previous chemotherapy regimens in the experimental arm was 3 (range 0–11). Investigators reported that olaparib led to a significantly longer PFS than placebo (8.4 vs. 4.8 months, HR 0.35, 95% CI 0.25–0.49, *P* < 0.001) [60]. A subsequent pre-planned retrospective analysis showed that median PFS was even longer in patients with g/s*BRCA*mt (11.2 vs. 4.3 months, HR 0.18, 95% CI 0.10–0.31, P < 0.0001) (see Table 3) [61]. Although the study showed a significant improvement in overall survival (OS) in the primary analysis (HR 0.73, 95% CI 0.55–0.96, *P* = 0.025) particularly in women with g/s*BRCA*mt (HR 0.62, 95% CI 0.41–0.94, *P* = 0.025), these results are considered descriptive as the trial was not originally powered to detect a difference in OS [64, 65].

### Phase 3 maintenance treatment trials

Three randomised, placebo-controlled, double-blinded phase 3 trials have investigated PARPi as maintenance treatment in women with ovarian cancer (see Table 3) [59, 62, 63]. All patients recruited had recurrent, platinum sensitive (defined as a progression-free interval of 6 months or more from the penultimate platinum chemotherapy) high-grade serous or endometrioid ovarian cancer and had achieved a CR/PR to their last platinum-based chemotherapy. Approximately 60% of patients had received two previous lines of chemotherapy before study enrolment and 60% had a PFI of at least 12 months. All participants were enrolled within 8 weeks of their last cycle of platinum.

In SOLO2/ENGOT-Ov21 (see Table 3: olaparib vs. placebo), only women with a g/s*BRCA*mt were eligible to participate, although no women with a somatic *BRCA* mutation were enrolled [63]. The dose and formulation of olaparib assessed in SOLO2 (300 mg twice daily in 100 or 150 mg tablets) differed from Study 19 (400 mg twice daily in 50 mg capsules) following phase 1/2 data showing improved bioavailability with 300 mg twice daily in a tablet formulation compared to 400 mg twice daily in a capsule formulation [49]. In ENGOT-OV16/NOVA [62] (see Table 3: niraparib vs. placebo), eligible patients had less than 2 cm of residual disease and normalisation of CA-125 following the last cycle of platinum therapy. In ARIEL3 [59] (rucaparib vs. placebo) no restrictions were made regarding the volume of residual disease following the last cycle of platinum chemotherapy and women with any persistent lesion greater than 2 cm diameter were defined as having bulky disease (rucaparib arm 19%; placebo 15%).

All three trials reported a significant improvement in the primary outcome of median PFS in women receiving PARPi compared to placebo (see Table 3). NOVA reported the longest PFS in the experimental arm (21.0 months; BICR PFS). In addition, in SOLO2 and ARIEL3 additional anti-tumour responses were reported beyond those achieved with the latest platinum therapy, confirming the cytotoxic effect of PARPi demonstrated in single-agent therapeutic trials (see Table 5) [58, 59].

**Table 5:** Objective response rates (ORR) as per RECIST criteria (CR/PR) for PARPi as maintenance treatment

Clinical Trial	PARP inhibitor	Subgroups	ORR, % (*n*)
Study 19	Olaparib 400 mg BD vs. placebo	HGSOC	12 (7/57) vs. 4 (2/48)
SOLO2	Olaparib 300 mg BD vs. placebo	g*BRCA*mt	41 (30/73) vs. 17 (6/35)
ARIEL3	Rucaparib 600 mg BD vs. placebo	g/s*BRCA*mt	38 (15/40) vs. 9 (2/23)
		HRD carcinoma	27 (23/85) vs. 7 (3/41)
		ITT population	18 (26/141) vs. 8 (5/66)

*BD* twice daily, *g* germline, *g/s* germline or somatic, *HRD* Homologous Recombination Deficiency, *ITT* intention-to-treat

The most encouraging results from NOVA and ARIEL3 were that cohorts of women with or without a g/s*BRCA*mt achieved a significantly improved PFS compared with patients who received placebo. Niraparib and rucaparib improved the median PFS by 5.4 months in the non-germline *BRCA1*/*2* mutation group (NOVA) and the intention-to-treat (ITT) population (ARIEL3) demonstrating the efficacy of the drugs in a genetically unselected population (see Table 3).

The data from these trials, in combination with the primary analysis from Study 19, demonstrate that PARPi are likely to benefit women with recurrent, platinum-sensitive high-grade serous ovarian cancer with or without a g/s*BRCA*mt. As all participants in these trials had platinum-sensitive disease this may be an appropriate and broader predictive biomarker to screen eligibility for PARPi.

There were some initial concerns that PARPi maintenance therapy may lead to platinum-resistance [66–70]. In those phase 3 trials with mature data, the median TFST and TSST were significantly longer in women treated with PARPi compared to placebo (see Table 4) [59, 61, 63, 71]. The TFST in women with a g*BRCA*mt was consistently in excess of 12 months across trials. Furthermore, the significant improvement in TSST across trials provides a surrogate marker of improved OS before full maturation of data. Thus, overall, there are no significant concerns that PARPi treatment induces platinum-resistance in a clinical setting.

As maintenance therapy, PARPi class-based AEs of fatigue, nausea, vomiting and anaemia were again evident. There was noticeably higher grade 3–4 neutropenia, thrombocytopenia and hypertension in patients receiving niraparib and grade 3–4 increases in ALT/AST in patients receiving rucaparib (see Table 6). The incidence of MDS/AML was around 1% across all PARPi. Safety data were not reported for individual cohorts of women and so it is unclear if the incidence and severity of off-target AEs was worse in women with a g*BRCA*mt vs. s*BRCA*mt or *BRCA*wt. Adverse events appeared to be manageable with dose reductions/interruptions and did not significantly affect quality of life [72–75].

**Table 6:** Key treatment-related adverse events in phase 3 trials assessing PARPi as maintenance treatment

	SOLO2 Olaparib 300 mg BD		NOVA Niraparib 300 mg OD		ARIEL3 Rucaparib 600 mg BD	
	G1-2 (%)	G3-4 (%)	Any grade (%)	G3-4 (%)	G1-2 (%)	G3-4 (%)
*Haematological adverse events*						
Anaemia	24	19	50	25	19	19
Neutropenia	14	5	30	20	11	7
Thrombocytopenia	13	1	61	34	13	5
*Gastrointestinal adverse events*						
Nausea	73	3	74	3	72	4
Vomiting	35	3	34	2	33	4
Diarrhoea	32	1	19	< 1	31	1
Abdominal pain	22	3	23	1	27	2
Constipation	21	0	40	< 1	35	2
Dyspepsia	11	0	11	0	14	< 1
*Other key adverse events*						
Fatigue	62	4	59	8	63	7
Dysgeusia	27	0	10	0	39	0
Headache	25	1	25	< 1	18	< 1
Decreased appetite	22	0	25	< 1	23	1
Dyspnea	11	1	19	1	13	0
Insomnia	6	0	24	< 1	14	0
Hypertension	3	0	19	8	NR	NR
Increase ALT/AST	5	3	10	4	23	10
Increase blood creatinine	11	1	NR^a^	NR^a^	15	< 1

*ALT* alanine aminotransaminase, *AST* aspartate aminotransaminase, *BD* twice daily, *NR* not reported, *OD* once daily

^a^FDA label for Niraparib states increase in blood creatinine identified in ≥ 1% to < 10% participants but grades not reported

Ongoing trials are also assessing the use of PARPi maintenance treatment as part of first-line chemotherapy as well as re-challenge PARPi maintenance treatment (see Table 2) [76–78].

## Genetic biomarkers

The clinical development of PARPi was predicted on the need for functional PARP1/2 in the absence of *BRCA1*/*2* function or other causes of homologous recombination deficiency (HRD). In keeping with these observations, genetic assays were investigated in NOVA and ARIEL3 to predict response to PARPi. Up to 50% of women with high-grade serous ovarian carcinoma may harbour a mutation in, or epigenetic silencing of, genes involved in HR repair [14, 79–84] and preclinical data suggested cells deficient in *BRCA1*/*2* and non-*BRCA* HR repair genes are sensitive to PARPi [6, 7, 10, 85, 86]. Other surrogate markers of HRD proposed as predictive biomarkers for PARPi include genetic mutations within the cancer genome suggestive of a greater reliance on alternative error-prone DNA damage repair mechanisms such as NHEJ and/or MMEJ [19, 87]. These putative biomarkers include genome-wide loss of heterozygosity (LOH), telomeric allelic imbalance (TAI) and large-scale state transitions (LST). Loss of heterozygosity is the irreversible loss of one parental allele [88]. Telomeric allele imbalances are regions of allelic imbalance that extend to one of the subtelomeres but do not cross the centromere [89]. Large-scale state transitions are chromosomal breaks between two adjacent regions of at least 10 Mb after filtering out regions less than 3 Mb [90]. Both *BRCA1*/*2* deficiency [89, 91] and sensitivity to platinum therapy [90] have been associated with greater LOH and increased regions of TAI and LST.

Next generation sequencing (NGS) assays can detect mutations in non-*BRCA* HR repair genes as well as the presence of LOH, TAI and/or LST in DNA extracted from tumour tissue. Two of these assays were prospectively evaluated in NOVA and ARIEL3, including Myriad myChoice® HRD test [62] (Myriad Genetics, Inc., UT, US) and Foundation Medicine T5 NGS assay [59] (Foundation Medicine, Inc., MA, US) respectively. The comparative results of these two technologies are presented below. These NGS-based assays may determine the presence/absence of mutations in genes involved in HR repair but do not provide information on function [92, 93].

### Myriad myChoice® HRD test

Myriad’s myChoice^®^ HRD test was assessed in NOVA as a predictive biomarker for niraparib maintenance treatment [62]. In women without a g*BRCA*mt (non-g*BRCA*mt), DNA was extracted from archival ovarian tumour tissue and sequenced for somatic mutations in *BRCA1*/*2* and 54,091 genome-wide single nucleotide polymorphisms (SNPs) to quantify LOH, TAI and LST [62]. In this assay, genomic LOH was specifically defined as the number of LOH regions greater than 15 Mb within the tumour genome, but less than a whole chromosome [91]. The assay calculates an algorithmic score (range 0 to 100) based on the accumulation of LOH, TAI and LST and produces a HRD “status”. All patients with a s*BRCA*mt and/or an algorithmic score of 42 or above were classified as HRD-positive, suggestive of HR deficiency [94, 95]. Patients with non-g*BRCA*mt and HRD-positive were enrolled into an independent cohort for the primary outcome analysis of BICR PFS (see Table 3). In addition, investigators assessed the primary outcome in all women with non-g*BRCA*mt including those with s*BRCA*mt, HRD-positive and HRD-negative tumour tissue (see Table 3).

### Foundation Medicine T5 NGS assay

The Foundation Medicine T5 NGS assay was assessed in ARIEL3 as a predictive biomarker for rucaparib maintenance treatment [59]. This assay was used to detect mutations in 30 HR repair genes and to quantify genome-wide LOH (as a percentage) by sequencing over 3500 evenly distributed SNPs in DNA extracted from archival ovarian tumour tissue [96]. The assay was unable to differentiate between g/s*BRCA*mt so blood could also be tested for germline mutations using the BRACAnalysis CDx test (Myriad Genetics, Inc., UT, US). Any patient found to have a g/s*BRCA*mt or genomic LOH of 16% or more were grouped together in a predefined cohort called “HRD carcinoma” for assessment of the primary outcome of investigator-assessed PFS (see Table 3). The cut off of 16% for LOH was determined following a retrospective analysis of ARIEL2 (Part 1) in which a clinically significant benefit in PFS was achieved in patients treated with rucaparib single-agent therapy who had at least 16% genomic LOH [53, 97]. The trial investigators in ARIEL3 also assessed the primary outcome in the ITT population, including all patients with either a g/s*BRCA*mt or *BRCA* wild-type/LOH^High^ or *BRCA* wild-type/LOH^Low^ (see Table 3).

Neither the Myriad myChoice® HRD test nor Foundation Medicine T5 NGS assay could fully predict which patients with relapsed, platinum-sensitive high-grade serous ovarian cancer were likely to benefit from niraparib or rucaparib maintenance therapy, respectively (see Table 3). The results from these trials raise important questions about the utility of genetic assays as predictive biomarkers in this selected group of patients. Both trials showed a tiered treatment effect, with the greatest benefit in PFS achieved in women with a g*BRCA*mt, however they also demonstrated that most women with platinum-sensitive high-grade serous ovarian carcinoma benefit from maintenance PARPi. This suggests that a conceptually simpler and much less expensive predictive biomarker for PARPi therapy could be platinum sensitivity.

The clinical relevance of possessing a germline or somatic mutation in a non-*BRCA* gene involved in HR repair as a predictive biomarker for PARPi also remains unclear. Data from both ARIEL2 (Part 1) and ARIEL3 reported around 10% of patients with high-grade serous ovarian cancer had a germline or somatic mutation in a non-*BRCA* HR repair gene [53, 59]. This represents a potential sizeable target population. Nevertheless, between these trials only patients with germline/somatic *RAD51C* or *RAD51D* mutations consistently achieved treatment responses to rucaparib [53, 98]. Indeed, the presence of a mutation in *RAD51C* or *RAD51D* correlated with an ORR (as per RECIST [CR/PR] and/or CA-125) to single-agent therapy with rucaparib in ARIEL2 (Part 1) and also led to encouraging median PFS [rucaparib: 16.4 months (range 5.4–30.4 months) versus placebo: 5.4 months (range 3.9–5.5 months)] with rucaparib maintenance treatment [53, 98]. Interestingly, all *RAD51C*/*D* mutations recorded in ARIEL2 (Part 1) and ARIEL3 correlated with the putative marker of genomic scarring “LOH^High^”, and, in ARIEL3 were reported as homozygous, suggesting biallelic LOF may bring about genomic instability and predispose to PARPi sensitivity. As a notable comparison approximately 80–90% of g*BRCA*mt carriers with ovarian cancer are reported to have LOH of the wild-type allele; in keeping with biallelic LOF and consistent with Knudson’s double hit hypothesis for tumour suppressor genes [23, 80, 82]. Therefore, if NGS-based assays are to be used on tumour tissue to determine eligibility for PARP inhibitors, it may be appropriate to consider screening for biallelic LOF (i.e. homozygosity) in *RAD51C* and *RAD51D* as well as *BRCA1* and *BRCA2*.

### Circulating tumour DNA as genetic biomarkers

Despite women with platinum-sensitive high-grade serous ovarian cancer achieving a significant PFS benefit to PARPi, the majority eventually develop progressive disease. A number of mechanisms of resistance to PARPi have been reported including intragenic reversions in the germline mutant alleles, loss of 53BP1, reduction in PAR expression and up-regulation of P-glycoprotein efflux pumps (reviewed in Lord et al. [99]). In clinical practice, the most frequently reported resistance mechanism is secondary reversion mutations/intragenic deletions in germline *BRCA1* [100, 101] or *BRCA2* [67–69, 100] mutations following platinum-based therapy or in germline *BRCA1* [102], *BRCA2* [66], *RAD51C and RAD51D* [102] mutations following PARPi therapy. The incidence of *BRCA1*/*2* reversion mutations in women with platinum-resistant ovarian cancer has been reported as high as 46% (95% CI 29–65%) [100]. In women with s*BRCA1*/*2* mutations, copy number alternation and up-regulation of the remaining wild-type allele has also been suggested as potential resistance mechanisms to PARPi [103]. Multiple reversions in germline mutations have been described in the same patient at different sites of metastatic disease as well as single biopsy sites [83, 102]. Reversion mutations have also been detected in circulating tumour DNA (ctDNA) in women who develop resistance to platinum [104–106] and in tumour tissue/ctDNA from patients with prostate and pancreatic cancer after progression following PARPi therapy [107–109]. Indeed, the use of temporal sampling of ctDNA may circumvent the challenges of determining differences in tumour heterogeneity and tumour evolution when analysing tumour tissue biopsies alone [110–112].

As yet, no prospective trial data are available regarding the incidence of reversion mutations in women with ovarian cancer who develop resistance to PARPi or their treatment responses to subsequent systemic anti-cancer therapy [70, 106]. Prospective trials are, therefore, required that utilise repeat tumour sampling and ctDNA to assess tumour evolution during therapy in women with g/s*BRCA*mt and *BRCA*wt ovarian cancer. In this respect, serial molecular analyses of tumour evolution should parallel traditional methods of determining progressive disease such as clinical, biochemical and radiological assessment, especially in those women with mutations in genes that may initially confer sensitivity to PARPi therapy and then potentially revert/mutant to bring about resistance.

## Conclusion

PARPi have anti-tumour activity as single agent therapy and offer effective maintenance treatment in recurrent, platinum-sensitive high-grade serous ovarian carcinoma, with the greatest benefits being demonstrated in women with g/s*BRCA*mt. Similar efficacy outcomes were demonstrated across a range of PARPi without any one drug showing superior efficacy. A number of class-based side effects have emerged, with early suggestions of particular drug-related side effects. Next generation sequencing-based assays that detect putative markers of HRD in tumour tissue or ctDNA, beyond, *BRCA1*/*2* mutational status, may have potential as predictive biomarkers in certain clinical settings but further research is required.
